# Detecting HER2-positive circulating tumor cells in gastric cancer using a flow cytometry-based approach

**DOI:** 10.3389/fmed.2025.1672955

**Published:** 2025-09-09

**Authors:** Jan-Mou Lee, Chia-Chun Tu, Chih-Hao Fang, Shian-Ren Lin, Shiu-Lan Wang, Wan-Yu Lai, Po-Wei Tseng, Wan-En Liao, Li-Yun Huang, Yee Chao, Ming-Huang Chen

**Affiliations:** ^1^FullHope Biomedical Co., Ltd., New Taipei City, Taiwan; ^2^Central Clinic and Hospital, Taipei City, Taiwan; ^3^Division of Gastroenterology and Hepatology, Department of Medicine, Taipei Veterans General Hospital, Taipei City, Taiwan; ^4^Department of Oncology, Taipei Veterans General Hospital, Taipei City, Taiwan; ^5^School of Medicine, National Yang Ming Chiao Tung University, Taipei City, Taiwan; ^6^Center of Immuno-Oncology, Department of Oncology, Taipei Veterans General Hospital, Taipei City, Taiwan

**Keywords:** circulating tumor cells, flow cytometry, gastric cancer, HER2 overexpression, liquid biopsy

## Abstract

**Background:**

HER2 overexpression predicts the response to trastuzumab in gastric cancer (GC); however, assessing this overexpression requires invasive immunohistochemistry. Circulating tumor cells (CTCs) share comparable proteomic phenotypes to those of the primary tumor and represent a less invasive method for evaluating HER2 expression. Nevertheless, methods for detecting HER2 expression on CTCs still require further investigation.

**Methods:**

A flow cytometry method for detecting HER2-overexpressed CTCs was established by spiking NCI-N87 cells into blood from cancer-naive donors. The method was then applied to blood samples from 14 gastric cancer (GC) patients (4 with HER2-overexpressed tumors and 10 with HER2-wild type tumors) and 10 cancer-naive participants (CNPs), with all data analyzed using one-way ANOVA.

**Results:**

The gating strategy was defined as CD45^−^CK-7/8^+^CK-14/15/16/19^+^EpCAM^+^HER2^+^, with a cut-off value of 5 cells/mL. Using this in-house method, we detected HER2-overexpressed CTCs in 4 of 4 GC patients with HER2-overexpressed tumors (ranging from 8 to 29 cells/mL), while 10 of 10 CNPs had undetectable HER2-overexpressed CTCs. Among GC participants with HER2-wild type tumors, 7 of 10 GC participants had undetectable HER2-overexpressed CTCs, whereas 3 of 10 had detectable HER2-overexpressed CTCs. Furthermore, one participant with HER2-overexpressed CTCs detected by our in-house method received HER2-based targeted therapy and experienced an objective response and was free from disease progression to the date of article writing.

**Conclusion:**

This study introduces a noninvasive method for monitoring HER2 expression in GC, offering new insights into providing personalized treatment strategies.

## 1 Introduction

According to the Global Cancer Observatory, 968,350 newly diagnosed cases of gastric cancer (GC; 4.9% of all cancer cases) and 659,853 GC-related deaths (6.8% of all cancer-related deaths) were in 2022, making GC is the fifth most common and lethal cancer worldwide (1). Because symptoms are often subtle or nonspecific, more than 60% of GC cases are diagnosed at advanced stages (2). Since approximately 21% of patients with GC carry HER2-overexpressed tumors (3), and HER2-overexpressed tumors are more aggressive than HER2-wild type tumors (4, 5), HER2-based targeted therapies, such as trastuzumab (brand name Herceptin) and its derivatives, pertuzumab (brand name Perjeta), and zanidatamab (brand name Ziihera), are considered as frontline treatment for those patients with HER2-overexpressed GC (6, 7).

According to clinical guidelines, HER2 expression in tumor tissues is assessed by either immunohistochemical staining (IHC)-based or *in situ* hybridization (ISH)-based companion diagnostics (CDx). Both methods have demonstrated high sensitivity and specificity in detecting HER2 expression in GC tumors (8–12). However, these CDx are performed only on samples obtained through solid biopsies or surgeries. Considering that in 30% of GC cases, intra-tumoral HER2 expression alters due to HER2-based targeted therapies (13), the timely monitoring of HER2 expression during treatment for fine-tuning regimens is critical, but this is currently impossible. Furthermore, approximately 5 to 69% of GC cases exhibit HER2 heterogeneity, which may cause underestimation of clinical response to HER2-based targeted therapies in those patients with heterogenous HER2 expression during clinical assessment (14). Additionally, the objective response rate of HER2-targeted therapies to those patients with HER2-overexpressed GC was ranged from 26.3 to 57.0% (15, 16), suggesting that intra-tumoral HER2 expression cannot fully correlate with clinical response to HER2-based targeted therapies. Hence, a rapid, less-invasive, accurate approach for assessing clinical response to HER2-based targeted therapies remains urgent but unmet.

Circulating tumor cells (CTCs) are tumor cells derived from primary tumors that can be categorized as epithelial (EpCAM^+^Vimentin^−^), mesenchymal (EpCAM^−^Vimentin^+^), and hybrid (EpCAM^+^Vimentin^+^) types (17). CTCs circulate in peripheral blood, and their genomic and proteomic characteristics can be identified after enumeration with size selection, immune-based selection, or precipitation (18). Since CTCs share comparable genomic and phenotypic characteristics with primary tumors (19), they can serve as a surrogate marker of HER2 expression—either genomic or proteomic—to primary tumors (20). Currently, the Food and Drug Administration of the United States has approved the CellSearch system for enumerating CTCs for further genomic or proteomic investigation, but this approval is only for breast cancer, lung cancer, and colorectal cancer (21), indicating that methods for detecting HER2 expression status on GC CTCs remains lacking.

To this end, we aimed to develop a protocol for detecting HER2-overexpressed CTCs using flow cytometry. We spiked NCI-N87 (N87, a GC cell line with HER2 overexpression) cells into peripheral blood samples from cancer-naïve donors for setting up the gating strategy and cut-off value. After method development, we used this in-house method to detect HER2-overexpressed CTCs from participants with GC and cancer-naïve participants (CNPs), and to evaluated the concordance of the results between our in-house method and IHC/ISH methods from tumor biopsy.

## 2 Materials and methods

### 2.1 Cells, reagents, and antibodies

In this study, NCI-N87 (N87; human gastric carcinoma cell line; BCRC 60217) cells were used as the model system. The cells were obtained from the Bioresource Collection and Research Center (Hsinchu, Taiwan). The cultivation of N87 cells followed the protocol provided by the vender. Briefly, the cells were cultured in RPMI 1640 (ATCC modified) medium (Thermo Fisher Scientific, Waltham, MA, USA) supplemented with 10% (v/v) fetal bovine serum (Thermo Fisher Scientific) and 1% (v/v) penicillin/streptomycin (Thermo Fisher Scientific). The culture medium was replaced every 2 to 3 days. When the cells reached 80% confluence, they were detached using TrypLE Select (Thermo Fisher Scientific) for further maintenance or scheduled experiments. All experiments were conducted within 10 passages.

The applied reagents and antibodies are listed in Tables 1, 2. All reagents and antibodies were aliquoted and stored in the condition recommended by the manufacturers until use.

**Table 1:** Reagents utilized in this study.

Name	Manufacture
For cell culture	
Fetal bovine serum	Thermo-Fisher
Penicillin/Streptomycin	Thermo-Fisher
ATCC-modified RPMI-1640	Thermo-Fisher
TrypLE select	Thermo-Fisher
For immunostaining	
Cell staining buffer	Biolegend
Foxp3 transcription factor staining buffer set	eBioscience
For Spike-in assay	
RBC lysis buffer	Biolegend

ATCC, American Type Culture Collection; RBC, red blood cell; RPMI-1640, Roswell Park Memorial Institute medium 1640.

**Table 2:** Antibodies utilized in this study.

Name	Clone	Fluorophore	Manufacture
For CTC identification			
CD45	J33	ECD	Beckman Coulter
Cytokeratin 7/8	CAM5.2	FITC	BD Pharmingen
Cytokeratin 14/15/16/19	KA4	AF647	BD Pharmingen
CD326 (EpCAM)	9C4	BV510	BioLegend
For phenotype analysis			
CD340 (erb-b2/Her2)	24D2	PE/Cy7	Biolegend
Isotype control			
Mouse IgG1	679.1Mc7	ECD	Beckman Coulter
Mouse IgG1 κ isotype	MOPC-21	PE/Cy7	BioLegend
		AF647	BD Pharmingen
Mouse IgG2a κ isotype	MOPC-173	FITC	BioLegend
Mouse IgG2b κ isotype	MPC-11	BV510	BioLegend

AF647, Alexa Fluor 647; BV510, Brilliant violet 510; Cy7, Cyanine 7; ECD, Electron coupled dye; FITC, Fluorescein isothiocyanate; PE, Phycoerythrin.

### 2.2 Erythrocyte lysis and immunostaining

Erythrocytes in peripheral blood samples were depleted by following the protocol from the manufacturer of RBC lysis buffer. Briefly, blood samples were mixed with 20 times the sample volume of RBC lysis buffer (Biolegend, San Diego, CA, USA). After incubation at room temperature, the mixtures were centrifuged at 400 × *g* for 10 min, and the remaining cells were collected in a pellet.

The immunostaining protocol was described in our previous study (22). Briefly, cells were suspended in cell staining buffer (BioLegend) and sequentially stained with antibodies targeting surface markers (CD45, EpCAM, and HER2) and intracellular markers (cytokeratins). Between the staining steps for surface and intracellular markers, the cells were fixed and permeabilized by using the Foxp3 transcription factor staining buffer set (eBiosciences, San Diego, CA, USA). After staining, the cells were analyzed for phenotypes by using a Gallios flow cytometer (Beckman Coulter, Brea, CA, USA). The positive gate for each fluorophore was set based on the staining of a matched isotype control.

### 2.3 Spike-in assay

The procedure of the spike-in assay referred to one literature with modification (23). Briefly, N87 cells were suspended in staining buffer and spiked into 1 mL of peripheral blood obtained from a CNP at concentrations of 2, 5, 10, and 25 cells/mL. The blood sample was then subjected to erythrocyte lysis, immunostaining, and fluorescence analysis.

### 2.4 Ethics statement

The current study protocol was designed in accordance with the Declaration of Helsinki. In addition, the Institutional Review Board of Taipei Veterans General Hospital reviewed and approved the protocol (approval codes 2021–12-003 AC and 2023–09-013CC).

### 2.5 Participant recruitment

Patients with GC and CNPs were recruited from Taipei Veterans General Hospital between October 2023 and December 2024. Individuals were considered for inclusion if they were aged over 20 years, histologically confirmed to have or not have HER2^+^ gastric carcinoma in accordance with clinical guidelines, did not have a diagnosis of acquired immunodeficiency syndrome or syphilis, and were not receiving any chemotherapy or radiotherapy within 1 month of blood collection. Individuals were excluded if they had confirmed metastasis in the central nervous system; pulmonary fibrosis or edema; severe and uncontrolled clinical illnesses in the cardiovascular, hepatic, or renal systems; or severe and uncontrolled infections. The inclusion and exclusion criteria for CNPs were the same as those for patients with cancer, excluding the requirement of confirmation for any types of cancer. Informed consent from patients with GC were obtained before they started treatment, and CNPs before blood collection. After obtaining informed consent, 10 mL of EDTA-anticoagulated peripheral blood was collected from the participants, shipped to the lab, proceeded to erythrocyte lysis, immunostaining, and fluorescence analysis.

### 2.6 Data acquisition and statistical analysis

Data were acquired and analyzed using Kaluza analysis software (Beckman Coulter). In addition to flow cytometry data analysis, data plotting and statistical analysis were performed using Prism (V9.5.1, GraphPad Software, Houston, TX, USA). Statistical significance was determined using the one-way ANOVA coupled with Kruskal-Wallis test. Comparison with statistical significance (*p* < 0.001) were labeled with ***.

## 3 Results

### 3.1 HER2-overexpressed CTC can be identified by a specific pedigree with a cut-off value of 5 cells/mL

**Figure 1:**
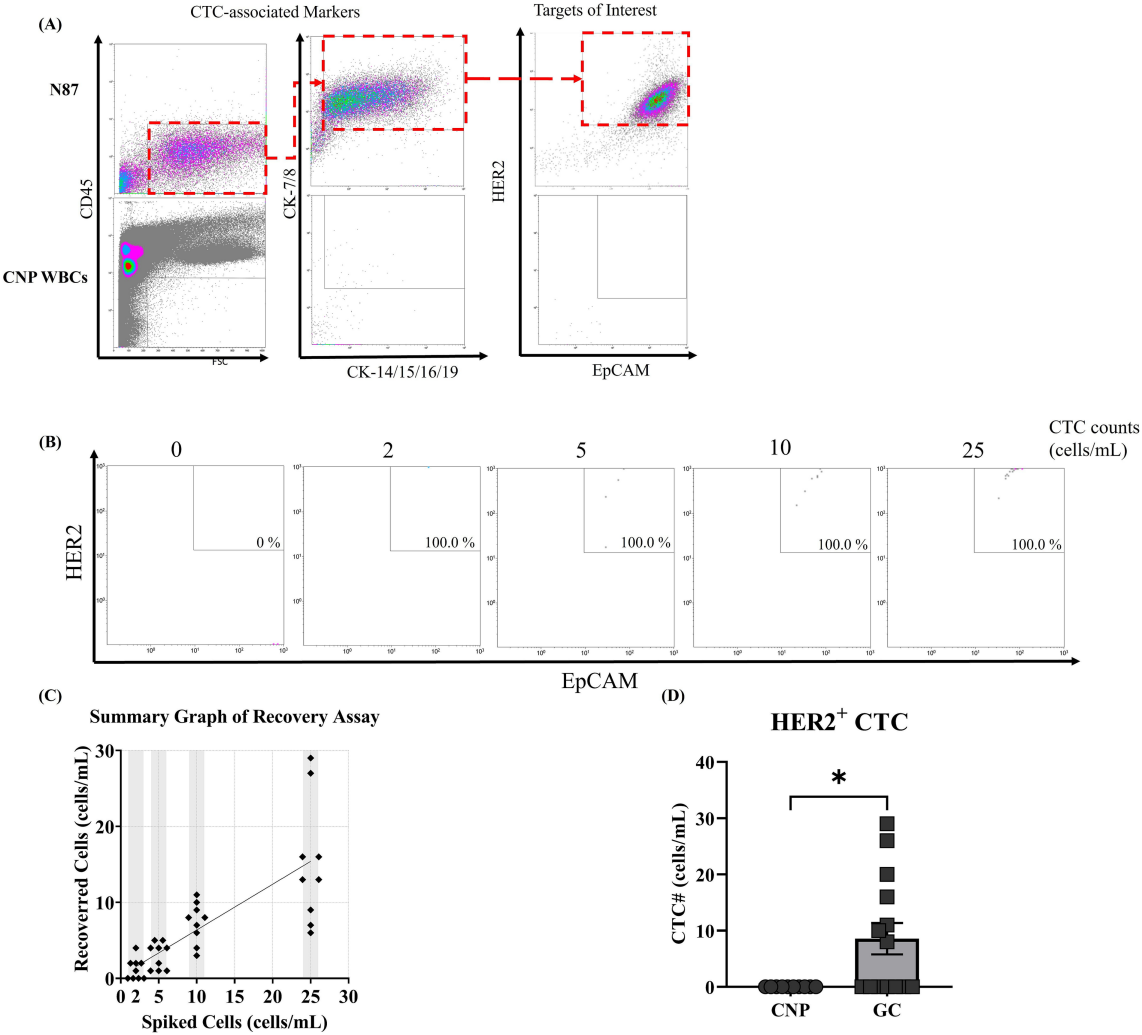
Establishment of a flow cytometry gating strategy and sensitivity for HER2-overexpressed circulating tumor cell detection. **(A)** NCI-N87 gastric cancer cells (N87) and leukocytes isolated from a cancer-naïve participant were immunostained with fluorescence-labeled antibodies targeting CD45, cytokeratins 7/8 (CK-7/8), cytokeratins 14/15/16/19 (CK-14/15/16/19), EpCAM, and HER2. Fluorescence signals were sequentially analyzed using forward scatter (FSC)/CD45, CK-7/8/CK-14/15/16/19, and EpCAM/HER2 parameters. **(B,C)** N87 cells were spiked into peripheral blood from a cancer-naïve participant (CNP) at concentrations ranging from 0 to 25 cells/mL. After immunostaining and flow cytometry, tumor cells were quantified using the CD45^−^/CK-7/8^+^/EpCAM^+^/HER2^+^ lineage, and the recovery rate between determined and spiked cell counts was measured. **(D)** Constructed flow pedigree able to correctly identify HER2-overexpressed CTCs from patients with HER2-overexpressed gastric cancer. The study recruited 14 patients with gastric cancer and 10 CNPs and collected their peripheral blood for the detection of HER2-overexpressed CTCs. The counts of HER2-overexpressed CTCs were summarized on the basis of their respective groups, and the mean counts were compared. Statistical significance was determined using the one-way ANOVA coupled with Kruskal-Wallis test, where comparison exhibited statistical significance (*p* < 0.001) were labeled with ***.

Firstly, we developed a gating strategy to identify HER2-overexpressed CTCs by flow cytometry. To this end, N87 cells were suspended in PBS, stained with CD45 (leukocyte marker), cytokeratin (CK-7/8, CK-14/15/16/19), EpCAM (epithelial cell marker), and trastuzumab (HER2-specific antibody) (24). Cellular fluorescence was detected by the flow cytometry. To access potential interference from blood cells, blood from a cancer-naïve donor were all included as a negative control. The majority of N87 cells were identified using CD45^−^CK-7/8^+^CK-14/15/16/19^+^EpCAM^+^HER2^+^ strategy, while no signal from blood cells were identified using the same strategy (Figures 1A,B). This indicated that CD45^−^CK-7/8^+^CK-14/15/16/19^+^EpCAM^+^HER2^+^ can be applied to detect HER2-overexpressed CTCs without interference from blood cells.

To evaluate the sensitivity and linearity of our in-house method, we spiked N87 cells into peripheral blood samples from a CNP at concentrations ranging from 2 to 25 cells/mL and counted HER2-overexpressed CTCs using our in-house method. Signals from N87 cells were found within the area we defined as HER2-overexpressed CTCs, with a 100% detection rate across the range of 2 to 25 cells/mL. This confirmed that our in-house method can detect HER2-overexpressed CTCs in peripheral blood (Figure 1B). When plotting counted cell numbers against indicated spiked cell numbers, counted numbers were consistently lower than spiked number, with an overall recovery rate of 60% (Figure 1C). A linear regression analysis between counted and spiked cell numbers produced the function y = 0.6024x + 0.3413, with an R^2^ value of 0.6158 (Figure 1C). This result indicates that our in-house method is suitable for qualitatively detect HER2-overexpressed CTCs rather than quantitative analysis. When analyzing the counted cell number in each spiked concentration, pseudo-negative results were observed at spiked cell numbers of 2 cells/mL but not at 5 cells/mL (Figure 1C). Based on this finding, we established a cut-off value with 5 cells/mL, where HER2-overexpressed CTC counts higher than this value were defined as detectable. In summary, our in-house method can qualitatively detect HER2-overexpressed CTCs in peripheral blood, with a cut-off value of 5 cells/mL. In the next section, we applied our in-house method to detect HER2-overexpressed CTCs in patients with GC and CNPs and compared results between our in-house method and IHC from tumor biopsy.

### 3.2 Developed pedigree could correctly detect HER2-overexpressed CTCs in patients with IHC-proven HER2-overexpressed GC

To evaluate the feasibility of our in-house method for detecting HER2-overexpressed CTCs in real-world samples, we collected peripheral blood from 14 patients with GC and 10 age-matched CNPs. The demographics of the participants are listed in Table 3. Among these patients, two were diagnosed with stage I, three with stage II, five with stage III, and four with stage IV. Analysis of HER2 expression in tumor biopsy indicated that 10 patients had a HER2 wild-type tumor (IHC score 0 or 1+) while four had HER2-overexpressed tumor (IHC score 3+) (10). At the time of enrollment, six (42.9%) participants received radical subtotal gastrectomy (two by laparoscopy) plus Billroth I gastroduodenostomy, four (28.9%) received radical subtotal gastrectomy (one by laparoscopy) plus Billroth II gastroduodenostomy, one (7.1%) received total gastrectomy plus roux-en-y esophagojejunostomy, one (7.1%) received laparoscopic proximal gastrectomy, and two (14.3%) did not received therapeutic surgery (one with feeding jejunostomy). HER2-overexpressed CTCs were undetectable in all samples from CNPs. In contrast, HER2-overexpressed CTCs were detectable in all samples from participants with HER2-overexpressed GC, with counts ranging from 8 to 29 cells/mL (Figure 1D). In samples from participants with HER2-wild type GC, three samples had detectable HER2-overexpressed CTCs (counts ranging from 10 to 20 cells/mL), while the remaining samples were undetectable (Figure 1D). The results in participants with HER2-overexpressed GC showed concordance between our in-house method and IHC, whereas the results from three participants with HER2-wild type GC showed discordance (Table 4). Considering that patients with HER2-wild type GC are not recommended to receive trastuzumab treatment (25), participants with HER2-wild type GC did not receive trastuzumab treatment. Nevertheless, one patient with discordant result between our in-house method and IHC received off-labeled trastuzumab and had an observable response at the time of writing. The following section presents a case abstract.

**Table 3:** Demographic of the participants.

Demographic	GC *N* = 14	CNP *N* = 10	*p* value
Male, *N* (%)	7 (50%)	6 (60%)	
Median age, years (range)	70 (79–50)	61.5 (74–49)	0.051
Stage, *N* (%)			
I	2 (14.3%)		
II	3 (21.4%)		
III	5 (35.7%)		
IV	4 (28.6%)		
Lymph node involvement, N (%)	6 (42.9%)		
Distal metastasis, *N* (%)			
Liver	2 (14.3%)		
Peritoneal	3 (21.4%)		
HER2 IHC score, *N* (%)			
0 or 1+	10 (71.4%)		
3+	4 (28.6%)		
Therapeutic surgery, N (%)	12 (85.7%)		
Ongoing treatment, *N* (%)			
Trastuzumab	2 (14.3%)		
ICIs^a^	4 (28.6%)		
Chemotherapy			
5-FU or tegafur	9 (64.3%)		
Capecitabine	2 (14.3%)		
Docetaxel	2 (14.3%)		
Folic acid	3 (21.4%)		
Gimeracil	6 (42.9%)		
Oteracil	6 (42.9%)		
Oxaliplatin	5 (35.7%)		
No treatment	3 (21.4%)		

5-FU, 5-fluorouracil; CNP, cancer-naïve participants; GC, gastric cancer; ICI, immune checkpoint inhibitors.

a ICIs applied in the enrolled patients included nivolumab and pembrolizumab.

**Table 4:** Detection performance comparison of HER2-overexpressed CTCs: flow cytometry vs. conventional testing.

		Flow cytometry	
		+	−
HER2 testing	3+	4	0
	0 or 1+	3	7

CTC, circulating tumor cell; HER2, epidermal growth factor receptor 2.

### 3.3 Case presentation

**Figure 2:**
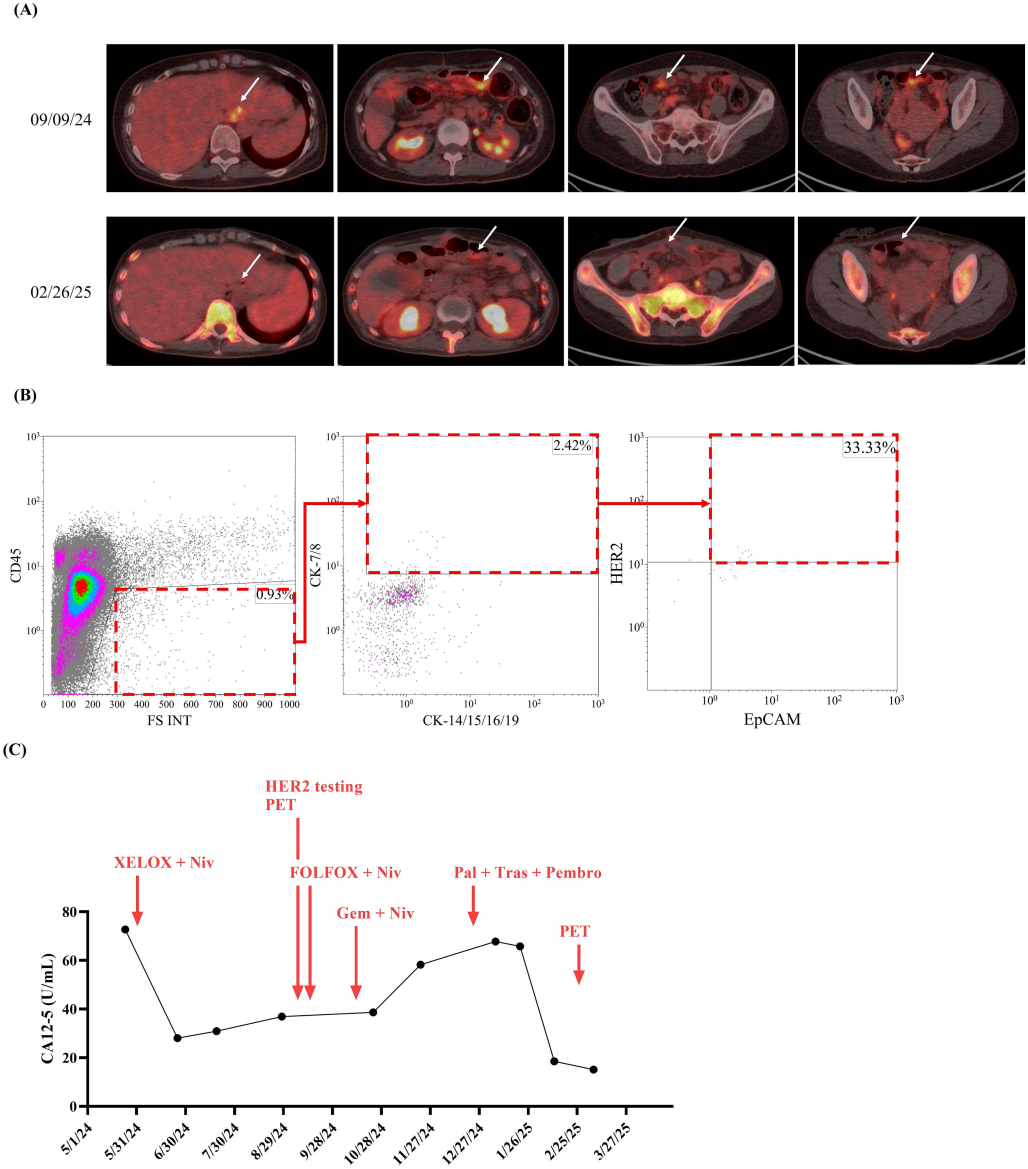
Time course, HER2-overexpressed CTC counts, and tumor imaging data of reported case. Since visiting Taipei Veterans General Hospital on May 24, 2024, cancer tumor markers, including **(A)** carbohydrate antigen 19–9 (CA19-9), **(B)** carcinoembryonic antigen (CEA), and **(C)** CA15-3, for the reported patient were monitored at least once every 3 months until March 20, 2025.

**Figure 3:**
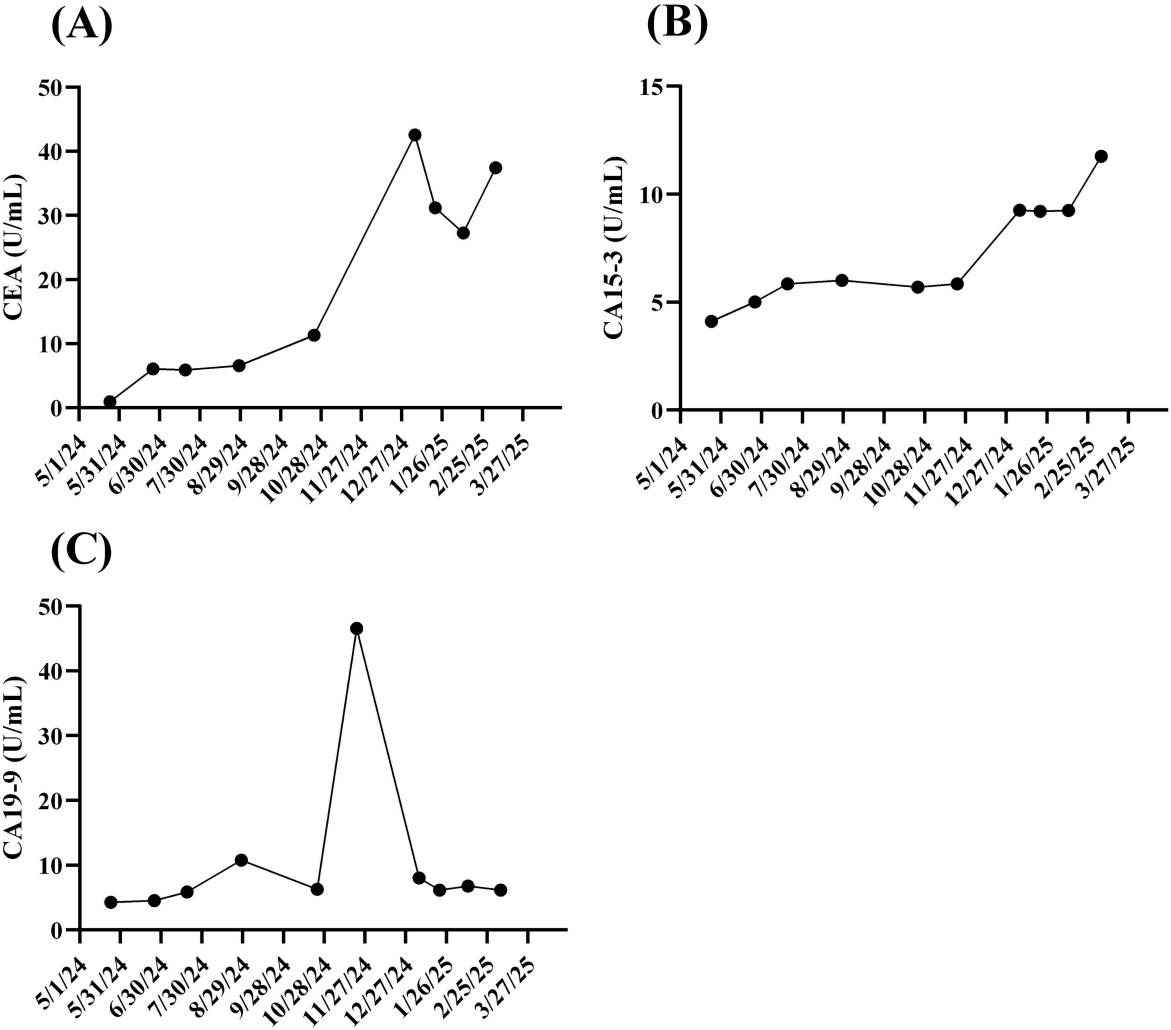
Time course of biochemical markers for gastric cancer. Since visiting Taipei Veterans General Hospital on May 24, 2024, cancer tumor markers, including 499 **(A)** carbohydrate antigen 19-9 (CA19-9), **(B)** carcinoembryonic antigen (CEA), and **(C)** CA15-3, for 500 the reported patient were monitored at least once every 3 months until March 20, 2025.

A 49-year-old woman presented to Taipei Veterans General Hospital on April 10, 2024. Whole-body positron emission tomography (PET) and a sono-hepatobiliary system evaluation revealed suspected tumor lesions in the stomach, with no significant findings in the liver or biliary system. The patient underwent laparoscopic radical total gastrectomy on April 23, 2024. Histopathological examination confirmed stage IIIA gastric adenocarcinoma (T4aN3b) with a linitis plastica phenotype. Immunohistochemical analysis of the resected tumor tissue revealed HER2 negativity (score 0). On May 27, 2024, the patient commenced adjuvant chemotherapy with XELOX (capecitabine plus oxaliplatin) and nivolumab. On September 9, 2024, refractory disease was diagnosed based on the identification of a new peritoneal lesion on routine PET imaging accompanied by this accompanied by increased carcinoembryonic antigen (CEA) and CA12-5 levels (Figure 2A, white arrow; Figures 3A,B). This confirmed disease progression to stage IV. In response, the chemotherapy regimen was switched to FOLFOX (leucovorin plus 5-fluorouracil and oxaliplatin) combined with nivolumab and ramucirumab on September 18, 2024. However, the patient experienced severe adverse events, including acute pancreatitis and purpura. Although the regimen was immediately switched to gemcitabine plus nivolumab, the severe adverse events persisted. Additionally, the CA19-9 level rapidly increased from 6.25 to 46.58 U/mL between October 23, 2024, and November 21, 2024 (Figure 3C). Consequently, treatment was suspended.

With informed consent, a liquid biopsy was performed on December 17, 2024, using our in-house method, which successfully identified HER2-overexpressing CTCs (Figure 2B). Based on this finding, an off-label treatment regimen comprising trastuzumab, pembrolizumab, and paclitaxel was initiated on December 25, 2024. On February 26, 2025, follow-up imaging revealed considerable shrinkage of the peritoneal lesion. Meanwhile, the serum CA12-5 level decreased from 67.79 to 18.50 U/mL (Figures 2A,C). Throughout this period (December 25 2024 to February 26 2025), CEA levels dropped from 42.58 to 27.28 U/mL, while CA19-9 levels remained stable, ranging between 6.13 and 8.01 U/mL. As of the date of manuscript preparation, the patient remains free from disease progression.

## 4 Discussion

In this study, we developed a flow cytometry–based method to detect HER2-overexpressing CTCs in the peripheral blood of GC patients. Our gating strategy, defined as CD45^−^CK-7/8^+^CK-14/15/16/19^+^EpCAM^+^HER2^+^, effectively identifies HER2-overexpressing CTCs in patients with an HER2 IHC score of 3+. Notably, this method also shows promise in identifying potential candidates for trastuzumab-based therapy among patients whose tumor biopsies have an IHC score of 0 or 1+. These results suggest that our in-house method is a valuable tool for clinicians to assess a patient’s eligibility for HER2-targeted therapy.

Trastuzumab-based therapies are recommended for patients with HER2-overexpressed GC (26, 27). During treatment, trastuzumab has been shown to induce a decrease in HER2 expression (13, 28). Timely monitoring of HER2 expression dynamics during trastuzumab therapy and fine-tuning regimens for—for example, switching to trastuzumab deruxtecan or trastuzumab emtansine—may help physicians improve treatment efficacy and reduce adverse events (29, 30). However, HER2 testing through IHC or ISH requires invasive tumor biopsies, which limits its routine clinical use during therapy. Therefore, a less invasive method for accurately assessing HER2 expression dynamics during treatment is required (31). Since CTCs share similar phenotypes with primary tumors (32), and the phenotyping of CTCs can be conveniently achieved through flow cytometry (33, 34), developing flow cytometry-based approaches to detect HER2 expression on CTCs holds promise as a surrogate for IHC or ISH assays of primary tumors.

According to the practical guideline, trastuzumab and its derivatives are recommended as a frontline treatment for patients with HER2-overexpressed GC (8, 35). Even though physicians typically determine HER2 expression before initiating therapy, a significant portion of patients with HER2-overexpressing GC do not receive trastuzumab at the start of treatment (36). Furthermore, intratumoral HER2 expression is not static and may change during the course of treatment, as it can be significantly altered by chemotherapy (37) or by HER2-based targeted therapy itself (38, 39). Given that repeated tumor biopsies are invasive and not suitable for routine monitoring, a practical alternative for physicians to continuously evaluate intratumoral HER2 expression remains an important but unmet clinical need. Our in-house method assesses HER2-overexpressing CTCs, providing a promising non-invasive tool to meet this unmet need. This approach can support physicians in determining if HER2-based targeted therapy is a feasible option for patients.

In addition to the in-house method presented in the present study, various approaches have been developed that utilize peripheral components to detect the HER2 phenotypes of primary tumors. Shoda et al. used droplet digital polymerase chain reaction and determined that the HER2 amplification status in circulating tumor DNA (ctDNA) is highly concordant with that of primary tumors, with a specificity of 0.733 and a sensitivity of 0.933 (40). These findings suggest that ctDNA can be used as a surrogate marker for local HER2 expression in primary tumors (40). In CTCs, HER2 expression can be detected using the CellSearch System (an FDA-approved CTC-analyzing system) or microfluidic chips coupled with fluorescence-labeled anti-HER2 antibodies or HER2-specific DNA probes (21, 41, 42). These studies have revealed that HER2 expression between CTCs and primary tumors is generally concordant; however, some patients with HER2-overexpressed CTCs exhibit HER2-wild type primary tumors. Treatment with trastuzumab in such patients has been reported to offer survival benefits (41). In our study, some patients with GC who tested negative for HER2 (testing score 0 or 1+) had detectable HER2-overexpressed CTCs. One of these patients achieved favorable clinical outcomes after trastuzumab treatment, implying that CTCs may provide complementary information for predicting trastuzumab response when traditional IHC or ISH assays of primary tumors yield negative results. Our in-house method uses CTCs as target to assess HER2 expression of primary tumors, a principle similar to the CellSearch system. However, CellSearch system is a CTC-enumerating system which requires additional method to analyze phenotypes on CTCs. Our in-house method is designed to directly detect HER2 expression status on CTCs, which streamlines the workflow and is more convenient for clinical application compared to other platforms that require a separate analysis step.

Although this study reports a promising method for detecting HER2-overexpressed CTCs in patients with GC, several limitations hinder its broader application. First, the correlation between HER2-overexpressed CTC counts and clinical response to trastuzumab remains unclear. This is despite the fact that one patient with HER2-overexpressed CTCs but wild type HER2 in IHC was reported to exhibit a clinical response to trastuzumab (41). Therefore, the relationship between the presence of HER2-overexpressed CTCs and the clinical benefit of trastuzumab treatment warrants further investigation. Second, the developed assay currently allows for only qualitative detection of HER2-overexpressed CTCs in patients with GC. Given that CTC counts are correlated with treatment response in various cancers (43, 44), the number of HER2-overexpressed CTCs may also correlate with the response to HER2-targeted therapies (45).

## 5 Conclusion

In conclusion, this study reports a promising novel flow cytometry-based method for the qualitative detection of HER2-overexpressed circulating tumor cells (CTCs) in patients with gastric cancer. The developed assay addresses a critical clinical challenge by offering a less invasive approach to assess HER2 status, particularly in cases where conventional tissue biopsies may yield negative results. Further investigations and an interdisciplinary collaboration among experts in various fields are warranted to establish the definitive clinical utility of this assay. The advancement of liquid biopsy technologies like this holds the potential to improve patient selection, guide treatment adjustments, and ultimately enhance clinical outcomes for individuals with gastric cancer.

## Data Availability

The raw data supporting the conclusions of this article will be made available by the authors, without undue reservation.
